# Risk factors for serious morbidity, prolonged length of stay and hospital readmission after laparoscopic appendectomy - results from Pol-LA (Polish Laparoscopic Appendectomy) multicenter large cohort study

**DOI:** 10.1038/s41598-019-51172-2

**Published:** 2019-10-15

**Authors:** Maciej Walędziak, Anna Lasek, Michał Wysocki, Michael Su, Maciej Bobowicz, Piotr Myśliwiec, Kamil Astapczyk, Mateusz Burdzel, Karolina Chruściel, Rafał Cygan, Wojciech Czubek, Natalia Dowgiałło-Wnukiewicz, Jakub Droś, Paula Franczak, Wacław Hołówko, Artur Kacprzyk, Wojciech Konrad Karcz, Jakub Kenig, Paweł Konrad, Arkadiusz Kopiejć, Adam Kot, Karolina Krakowska, Maciej Kukla, Agnieszka Leszko, Leszek Łozowski, Piotr Major, Wojciech Makarewicz, Paulina Malinowska-Torbicz, Maciej Matyja, Maciej Michalik, Adam Niekurzak, Damian Nowiński, Radomir Ostaszewski, Małgorzata Pabis, Małgorzata Polańska-Płachta, Mateusz Rubinkiewicz, Tomasz Stefura, Anna Stępień, Paweł Szabat, Rafał Śmiechowski, Sebastian Tomaszewski, Viktor von Ehrlich-Treuenstätt, Maciej Wasilczuk, Mateusz Wierdak, Anna Wojdyła, Jan Wojciech Wroński, Leszek Zwolakiewicz, Michał Pędziwiatr

**Affiliations:** 1 Department of General, Oncological, Metabolic and Thoracic Surgery, Military Institute of Medicine, Warsaw, Poland; 2 2nd Department of General Surgery, Jagiellonian University Medical College, Krakow, Poland; 3 Department of Surgical Oncology, Medical University of Gdansk, Gdansk, Poland; 4 1st Department of General and Endocrinological Surgery, Medical University of Bialystok, Bialystok, Poland; 5 2nd Department of General, Vascular and Oncological Surgery, Second Faculty of Medicine, Warsaw, Poland; 6 Department of General Surgery, SPZOZ in Węgrów, Węgrów, Poland; 7 Department of General, Oncological and Minimal Invasive Surgery, Żeromski’s General Hospital, Krakow, Poland; 8 Department of General, Minimally Invasive and Oncology Surgery, Regional Hospital named J.Śniadecki, Białystok, Poland; 9 Department of General, Minimally Invasive and Elderly Surgery, University of Warmia and Mazury in Olsztyn, Olsztyn, Poland; 10 Department of General and Oncological Surgery, Ceynowa Hospital, Wejherowo, Poland; 11 Department of General, Transplant and Liver Surgery, Medical University of Warsaw, Warszawa, Poland; 12 Clinic of General, Viscera and Transplantation Surgery, Ludwig Maximilian University, Munich, Germany; 13 Department of General, Oncologic and Geriatric Surgery, Jagiellonian University Medical College, Krakow, Poland; 14 Department of General Surgery and Surgical Oncology, Specialist Hospital in Kościerzyna, Kościerzyna, Poland; 15 Department of General, Oncological and Vascular Surgery, The Regional Subcarpathian John Paul II Hospital in Krosno, Krosno, Poland; 16 Clinical Department of General Surgery with Oncology, Gabriel Narutowicz Memorial City Specialty Hospital, Krakow, Poland; 17 Department of General and Laparoscopic Surgery, Municipal Hospital in Hajnówka, Hajnówka, Poland; 18 Department of General Surgery, Multispeciality Hospital in Nowa Sól, Nowa Sól, Poland; 19 Department of General and Minimally Invasive Surgery, Leczna Hospital, Leczna, Poland; 20 Department of General Surgery, Oncological Surgery and Chemotherapy, Dr Louis Błażek Memorial Hospital, Inowrocław, Poland; 21 Faculty of Health Sciences, Powiślańska School in Kwidzyn, Kwidzyn, Poland; 22 Emergency Department, Specialist Hospital in Kościerzyna, Kościerzyna, Poland

**Keywords:** Caecum, Medical research, Comorbidities

## Abstract

Laparoscopic appendectomy (LA) for treatment of acute appendicitis has gained acceptance with its considerable benefits over open appendectomy. LA, however, can involve some adverse outcomes: morbidity, prolonged length of hospital stay (LOS) and hospital readmission. Identification of predictive factors may help to identify and tailor treatment for patients with higher risk of these adverse events. Our aim was to identify risk factors for serious morbidity, prolonged LOS and hospital readmission after LA. A database compiled information of patients admitted for acute appendicitis from eighteen Polish and German surgical centers. It included factors related to the patient characteristics, peri- and postoperative period. Univariate and multivariate logistic regression models were used to identify risk factors for serious perioperative complications, prolonged LOS, and hospital readmissions in acute appendicitis cases. 4618 laparoscopic appendectomy patients were included. First, although several risk factors for serious perioperative complications (C-D III-V) were found in the univariate analysis, in the multivariate model only the presence of intraoperative adverse events (OR 4.09, 95% CI 1.32–12.65, p = 0.014) and complicated appendicitis (OR 3.63, 95% CI 1.74–7.61, p = 0.001) was statistically significant. Second, prolonged LOS was associated with the presence of complicated appendicitis (OR 2.8, 95% CI: 1.53–5.12, p = 0.001), postoperative morbidity (OR 5.01, 95% CI: 2.33–10.75, p < 0.001), conversions (OR 6.48, 95% CI: 3.48–12.08, p < 0.001) and reinterventions after primary procedure (OR 8.79, 95% CI: 3.2–24.14, p < 0.001) in the multivariate model. Third, although several risk factors for hospital readmissions were found in univariate analysis, in the multivariate model only the presence of postoperative complications (OR 10.33, 95% CI: 4.27–25.00), reintervention after primary procedure (OR 5.62, 95% CI: 2.17–14.54), and LA performed by resident (OR 1.96, 95% CI: 1.03–3.70) remained significant. Laparoscopic appendectomy is a safe procedure associated with low rates of complications, prolonged LOS, and readmissions. Risk factors for these adverse events include complicated appendicitis, postoperative morbidity, conversion, and re-intervention after the primary procedure. Any occurrence of these factors during treatment should alert the healthcare team to identify the patients that require more customized treatment to minimize the risk for adverse outcomes.

## Introduction

One of the most common intraabdominal conditions requiring surgical intervention is acute appendicitis (AA). According to the Global Burden of Disease Study (2016), 114.44 to 481.60 surgeries were required per 100 000 incidences of AA, depending on the socio-economic level of countries^1^. The incidence of appendicitis in newly industrialized countries is rising rapidly^2^. Appendectomy is the standard treatment of choice for acute appendicitis^3,4^. Although open appendectomy (OA) performed through the right lower quadrant incision remained nearly unchanged for over a century because of its safety and efficacy, laparoscopic appendectomy (LA) has gradually gained acceptance. Recent studies show that laparoscopic appendectomy provides considerable benefits over open appendectomy, including a lower complication rate, a shorter length of hospital stay (LOS), less postoperative pain and earlier postoperative recovery^5,6^. Laparoscopy also was associated with lower surgical site infection (SSI) rates^7,8^.

As with any surgical procedure, however, LA is associated with some risk of unfavorable outcomes. In general, the rate of perioperative complications and the length of hospital stay are universal measures of quality of treatment. Special attention was also paid to the readmission rates. Despite the constant improvements in perioperative care and increasing popularity of laparoscopy, certain patients still develop complications after LA. Although these unfavorable postoperative courses occur rather rarely in appendectomy comparison to other abdominal procedures, patients with these outcomes suffer delayed recovery.

Although laparoscopic appendectomy is slowly becoming the gold standard in acute appendicitis treatment thanks to growing evidence from the results of randomized controlled trials comparing LA with open surgery, data from large cohorts is still lacking. Such analyses may reflect more realistic populations without the restrictions commonly present in clinical trials. Therefore, we designed a multicenter cohort study to analyze the risk factors for unfavorable postoperative outcomes: morbidity, prolonged length of stay and readmissions after LA.

## Methods

### Study design

A study was performed over a 6-month period involving 18 Polish and German surgical centers. A database compiled the data of patients admitted for acute appendicitis^9^. The local team of nurses, anesthesiologists, and assistants along with the coordinating surgeon acquired data in each participating surgical unit.

The database included following patient characteristics: sex, age, and body mass index (BMI). It also followed many operative metrics: type of acute appendicitis (uncomplicated or complicated as determined by the presence of gangrenous appendicitis or perforated appendix with or without abscess)^10^, intraoperative adverse events (IAE), and postoperative outcomes (postoperative morbidity, need for surgical reintervention, LOS, and need for readmission to the original surgical department for whatever reason).

It was built along several parameters: ASA score, history of smoking, diabetes mellitus (DM), timing from onset of symptoms to surgery, operative parameters. The study adhered to the guidelines of The Strengthening the Reporting of Observational Studies in Epidemiology (STROBE) statement^11^. Complicated AA was diagnosed relying imaging diagnostics and/or visualization during relaparoscopy/relaparotomy performed due to patient’s condition.

The Videosurgery Chapter of the Association of Polish Surgeons supported this project. No changes in treatment strategy were included in the study protocol. A chief investigator had the responsibility of monitoring this study, by verifying missing or unclear data entered into the database. The database was anonymous as any information that could possibly identify the patients was excluded. Due to the observational nature and lack of patient personal data the study did not need informed consent. Approval by the ethics committee has been obtained from each of the participating centers for conducting this study. The presence of major complications (Clavien-Dindo III-V)^12^ in the postoperative period, prolonged LOS (LOS equal or longer than 2*upper quartile was recognized as prolonged LOS) and the need for readmission within 30 days were analyzed in order to identify potential risk factors.

### Statistical analysis

Statistical analyses were done using Statsoft STATISTICA 13.0 PL (Statsoft Inc., Tulsa, Oklahoma, USA). Continuous variables were presented using means with standard deviations (SD) or medians with inter-quartile ranges (IQR) for skewed variables. Groups were compared using Kruskal-Wallis’ ANOVA test with multiple comparison of ranges. Then, comparisons between groups were done using t-student tests for normally distributed variables and Mann-Whitney’s tests for skewed variables. Dichotomous variables were included in chi-squared Pearson’s, Yates’, and Fisher’s exact tests, depending on the quantities in the subgroups. Finally, univariate and multivariate logistic regression models were built to determine odds ratios for risk factors depending on postoperative complications, prolonged LOS and need for readmission. Results were considered statistically significant when p-values were <0.05. In the case of missing data, pairwise deletion was used.

### Ethics approval and consent to participate in the study

Ethics approval of the ethics committee of Jagiellonian University Medical College was obtained (nr 1072.6120.204.2018 20.09.2018). The data was completely anonymized, and no patient or hospital information was collected in the database. The study protocol was approved by the board of the Videosurgery Chapter of the Association of Polish Surgeons, and the study was conducted under its supervision. All procedures have been performed in accordance with the ethical standards laid down in the 1964 Declaration of Helsinki and its later amendments (Fortaleza).

## Results

4618 patients qualified for laparoscopic appendectomy. Table 1 presents the demographic data for these patients.

**Table 1:** General group characteristics.

No (%)		4618 (100%)
Males/Females, n (%)		2409/2209 (52%/48%)
Median age, years (IQR)		33 (24–47)
Median BMI, kg/m^2^ (IQR)		24.8 (22.03–28.5)
ASA class	1	3214 (69.6%)
	2	1213 (26.27%)
	3	184 (3.98%)
	4	7 (0.15%)
Smoking, n (%)		794 (17.19%)
Diabetes mellitus, n (%)		147 (3.18%)
Symptoms <48 h vs. >48 h, n (%)		3152/1466 (68.25%/31.75%)
Median Alvarado score (IQR)		6 (5–8)
Median leukocytosis, *1000 per mm^3^ (IQR)		13.1 (10.04–16.1)
Median CRP, mg/l (IQR)		27.33 (6.2–72)
No. of laparoscopic appendectomies/year in department, n (%)	>50	1349 (29.21%)
	<50	3269 (70.79%)
First operator, n (%)	Specialist	2589 (56.06%)
	Resident	2029 (43.94%)
Median operative time, min (IQR)		55 (40–70)
Technique, n (%)	Clipping	2841 (61.64%)
	Suturing/ligature	453 (9.79%)
	Stapler	313 (6.75%)
	Endoloop	606 (13.11%)
	Röder loop	403 (8.71%)
Complicated appendicitis, n (%)		1269 (27.48%)
Uncomplicated appendicitis, n (%)		3349 (72.52%)
Intraoperative adverse events, n (%)		104 (2.25%)
Drainage, n (%)		3493 (75.64%)
Postoperative morbidity, n (%)		310 (6.71%)
Clavien-Dindo classification of surgical complications, n (%)	I	146 (3.16%)
	II	80 (1.73%)
	III	77 (1.67%)
	IV	4 (0.09%)
	V	3 (0.06%)
Conversions, n (%)		294 (6.37%)
Reinterventions after primary procedure, n (%)		98 (2.12%)
LOS		3 (2–4)
Readmissions, n (%)		118 (2.56%)

In terms of gender differences, the number of males, 2409 (52.2%), exceeded that of females, 2209 (47.8%). The median age of patients was 33 years (IQR 24–47). Median BMI was 24.8 (IQR 22.03–28.5). 794 (19.19%) were active smokers and 147 (3.18%) had diabetes mellitus. The majority of patients, 3214 (69.60%), were classified as ASA I patients. The remainder were sorted as such: 1213 (26.27%) patients as ASA II, 184 (3.98%) patients as ASA III, and 7 (0.15%) patients as ASA IV. In 1463 (31.68%) patients, symptoms of AA were present 48 hours before surgery. The number of patients with postoperative morbidity were 310 (6.71%); according to the Clavien-Dindo (C-D) classification of surgical complications there were 3 (0.06%) patients with class V complications, 4 (0.09%) class IV, 77 (1.67%) class III, 80 (1.73%) class II, and 146 (3.16%) class I. As for complications; there were surgical site infection (1.97%,), intraabdominal abscess (1.3%), ileus (0.54%), intraabdominal bleeding (0.41%), pneumonia (0.39%), urinary tract infection (0.39%), diffuse peritonitis (0.17%), deep vein thrombosis (0.06%), bowel perforation (0.04%), pulmonary embolism (0.02%) and other (1.41%). Median length of stay (LOS) in the entire cohort was 3^2–4^ days. Prolonged LOS (LOS ≥8 days) was necessary in 227 (4.92%) of them, whereas readmission was recorded in 110 (2.56%) cases.

In univariate regression models, factors were identified that increase the risk of perioperative serious complications (C-D III-V) whose parameters are presented in Table 2. When a multivariate model was built taking into consideration parameters significant in univariate calculations, only intraoperative adverse events (OR 4.09, 95% CI 1.32–12.65, p = 0.014) and complicated appendicitis (OR 3.63, 95% CI 1.74–7.61, p = 0.001) remained significant (Table 2).

**Table 2:** Risk factors for perioperative serious complications (C-D III-V).

	OR	95%CI	p-value
**Univariate**			
Males/Females	0.63	0.40–0.99	**0.045**
Age >50	2.08	1.32–3.26	**0.002**
Obesity	0.98	0.45–2.15	0.962
ASA with every grade higher	1.71	1.17–2.48	**0.005**
Smoking	1.28	0.69–2.39	0.428
Diabetes	3.24	1.46–7.20	**0.004**
Symptoms >48 h vs. <48 h	2.44	1.55–3.85	**<0.001**
Alvarado	1.13	1.01–1.26	**0.035**
Leukocytosis	1.02	1.00–1.04	**0.021**
Leukocytosis >20,000/mm^3^	2.48	1.42–4.33	**0.001**
CRP >100	3.87	2.39–6.27	**<0.001**
No of appendectomies/year in department	1.42	0.85–2.39	0.185
Specialist vs. resident	0.86	0.56–1.32	0.492
Operative time	1.01	1.01–1.02	**<0.001**
Operative time/10 min	1.12	1.06–1.19	**<0.001**
Clipping	0.68	0.44–1.05	0.086
Suturing/ligature	2.78	1.65–4.67	**<0.001**
Stapler	0.87	0.35–2.18	0.774
Endoloop	1.22	0.67–2.21	0.510
Röder loop	0.38	0.12–1.22	0.106
Complicated vs. uncomplicated appendicitis	3.80	2.45–5.90	**<0.001**
Intraoperative adverse events	4.20	1.89–9.336	**<0.001**
Conversions	4.03	2.33–6.98	**<0.001**
**Multivariate**			
Males/Females	0.66	0.35–1.26	0.208
Age >50	0.68	0.29–1.59	0.376
ASA with every grade higher	1.26	0.70–2.26	0.448
Diabetes	1.67	0.51–5.50	0.397
Symptoms >48 h vs. **<**48 h	1.61	0.84–3.09	0.153
Alvarado	1.10	0.95–1.27	0.223
Leukocytosis >20,000/mm^3^	2.02	0.88–4.63	0.098
CRP >100	1.83	0.93–3.61	0.081
Complicated vs. uncomplicated appendicitis	3.63	1.74–7.61	**0.001**
Intraoperative adverse events	4.09	1.32–12.65	**0.014**
Conversions	1.48	0.67–3.24	0.328

Table 3 shows the details of univariate logistic regression models for increases in the risk of prolonged LOS. In the later multivariate model, only complicated appendicitis (OR 2.8, 95% CI: 1.53–5.12, p = 0.001), postoperative morbidity (OR 5.01, 95% CI: 2.33–10.75, p < 0.001), conversions (OR 6.48, 95% CI: 3.48–12.08, p < 0.001) and reinterventions after primary procedure (OR 8.79, 95% CI: 3.2–24.14, p < 0.001) were statistically significant.

**Table 3:** Risk factors for prolonged LOS.

	OR	95%CI	p-value
**Univariate**			
Males/Females	0.75	0.57–0.98	**0.035**
Age	1.03	1.02–1.04	**<0.001**
Age >50	2.78	2.10–3.64	**<0.001**
BMI with every kg/m^2^	0.99	0.98–1.01	0.286
Obesity	0.98	0.67–1.44	0.928
ASA with every grade higher	1.67	1.33–2.10	**<0.001**
Smoking	1.29	0.89–1.88	0.179
Diabetes	3.96	2.45–6.40	**<0.001**
Symptoms **<**48 h vs. >48 h	1.72	1.29–2.29	**<0.001**
Alvarado	1.10	1.03–1.19	**0.008**
Leukocytosis	1.02	1.00–1.03	**0.020**
Leukocytosis >20,000/mm^3^	1.57	1.06–2.35	**0.026**
CRP	1.01	1.00–1.03	**0.002**
CRP >100	5.38	3.88–7.44	**<0.001**
No of appendectomies/year in department	0.73	0.55–0.97	**0.029**
Specialist vs. resident	1.47	1.11–1.94	**0.007**
Operative time	1.02	1.01–1.02	**<0.001**
Operative time/10 min	1.17	1.13–1.22	**<0.001**
Clipping	0.56	0.43–0.73	**<0.001**
Suturing/ligature	3.40	2.48–4.67	**<0.001**
Stapler	0.43	0.20–0.92	**0.029**
Endoloop	1.22	0.84–1.78	0.286
Röder loop	0.96	0.60–1.52	0.858
Complicated vs. uncomplicated	6.07	4.56–8.07	**<0.001**
Intraoperative adverse events	2.62	1.41–4.86	**0.002**
Postoperative morbidity	35.98	26.05–49.71	**<0.001**
Conversions	6.50	4.69–8.99	**<0.001**
Reinterventions after primary procedure	26.78	17.52–40.92	**<0.001**
Readmissions	3.52	2.06–6.02	**<0.001**
**Multivariate**			
Males/Females	1.72	0.99–2.96	0.053
Age >50	1.74	0.90–3.38	0.099
ASA with every grade higher	1.11	0.66–1.86	0.688
Diabetes	1.06	0.37–3.00	0.918
Symptoms **<**48 h vs. >48 h	1.12	0.64–1.99	0.686
Alvarado	1.06	0.93–1.21	0.389
Leukocytosis >20,000/mm^3^	1.02	0.45–2.28	0.964
CRP >100	1.41	0.77–2.57	0.263
No of appendectomies/year in department	1.33	0.63–2.82	0.454
Complicated vs. uncomplicated	2.80	1.53–5.12	**0.001**
Intraoperative adverse events	2.49	0.85–7.31	0.096
Postoperative morbidity	5.01	2.33–10.75	**<0.001**
Conversions	6.48	3.48–12.08	**<0.001**
Reinterventions after primary procedure	8.79	3.20–24.14	**<0.001**

In univariate logistic regression analysis following risk factors for hospital readmissions were identified and presented in Table 4. However, in multivariate regression, only postoperative complications (OR 10.33, 95% CI: 4.27–25.00), reintervention after primary procedure (OR 5.62, 95% CI: 2.17–14.54), and LA performed by resident (OR 1.96, 95% CI: 1.03–3.70) remained significant.

**Table 4:** Risk factors for hospital readmissions.

	OR	95%CI	p-value
**Univariate**			
Males/Females	1.08	0.74–1.58	0.694
Age	1.00	0.99–1.01	0.371
BMI with every kg/m^2^	1.01	1.00–1.02	**0.008**
Obesity	1.38	0.72–2.65	0.331
ASA with every grade higher	1.44	1.00–2.07	**0.049**
Smoking	1.10	0.65–1.84	0.732
Diabetes	1.92	0.83–4.47	0.129
Symptoms **<**48 h vs. >48 h	1.01	0.95–1.07	0.838
Alvarado	1.07	0.96–1.19	0.215
Leukocytosis	1.01	0.99–1.03	0.379
CRP >100	1.92	1.20–3.09	**0.007**
No of appendectomies/year in department	1.84	1.15–2.95	**0.011**
Specialist vs. resident	0.50	0.34–0.73	**<0.001**
Operative time	1.00	0.99–1.01	0.139
Clipping	0.92	0.63–1.35	0.680
Suturing/ligature	1.74	1.04–2.91	**0.035**
Stapler	0.33	0.05–2.42	0.278
Endoloop	0.97	0.64–1.48	0.896
Röder loop	0.66	0.30–1.43	0.290
Complicated vs. uncomplicated	1.81	1.22–2.66	**0.003**
Intraoperative adverse events	0.43	0.06–3.13	0.405
Postoperative morbidity	19.27	12.81–28.99	**<0.001**
Conversions	2.03	1.12–3.68	**0.019**
Reinterventions after primary procedure	43.00	26.85–68.87	**<0.001**
LOS	1.05	1.02–1.09	**0.003**
**Multivariate**			
ASA	0.99	0.55–1.78	0.964
CRP >100	1.13	0.52–2.43	0.759
>50 appendectomies/year in department	1.17	0.55–2.51	0.680
Specialist vs. resident	0.51	0.27–0.97	**0.039**
Complicated vs. uncomplicated	0.63	0.28–1.41	0.260
Perioperative morbidity	10.33	4.27–25.00	**<0.001**
Conversions	0.88	0.40–2.96	0.876
Reintervention	5.62	2.17–14.54	**<0.001**

## Discussion

In our study we identified risk factors for serious morbidity, prolonged length of hospital stay and hospital readmission after LA. The risk factors for serious morbidity were complicated appendicitis and IAE; for prolonged hospital stay, they were complicated appendicitis, postoperative morbidity, conversion, and reintervention after primary surgery; and for hospital readmission: operation performed by surgical resident, perioperative morbidity and reintervention were risk factors. Interestingly, complicated appendicitis was an independent risk factor for complications and prolonged hospital stay as well, but it was not associated with a higher hospital readmission rate.

Our study reported an overall complications rate of about 6%. It is comparable with data reported in large cohorts that allows objective analysis of our results^13–16^. Importantly, LA in our cohort is characterized by low mortality (0.06%) and by a very low percentage (1.82%) of severe morbidity expressed as Clavien Dindo III-V, which proves the safety of the procedure. Andert *et al*. reported similar data. In this study, 1.7% of patients presented with major complications^17^. In an Australian study, 2.7% presented with an intra-abdominal abscess and 1.5% patients needed further intervention^15^. Large cohorts showed higher mortality up to 0.6%^18–20^. Although univariate logistic regression models showed several risk factors for severe postoperative complications, in the multivariate model only the presence of complicated appendicitis (OR 3.63) and intraoperative adverse events (OR 4.09) proved to be an independent risk factors associated with postoperative morbidity. Tiwari *et al*. observed that the severity of appendicitis correlated with higher morbidity and mortality independent of whether OA or LA was performed^21^. What has to be emphasized is that some clinical conditions have been previously identified as related to complicated AA, such as the co-existence of diabetes mellitus, elevated CRP, and leukocytosis^22,23^. Moreover, complicated appendicitis is often the reason to conversion and longer operative time^24,25^. It seems, therefore, that complicated AA may be simply considered a different condition that has the most significant impact on outcomes.

This observation regarding complicated AA has also been confirmed when prolonged LOS was analyzed. This condition remained the only independent factor for delayed recovery, apart from factors that are inevitably associated with longer hospital stay such as conversion or complications. LOS frequently depends on the traditional attitude and model of perioperative care^26^. Median LOS in the entire cohort in our study was 3 days, similar to previous reports^19,26^. On the other hand, there are studies reporting LA in uncomplicated acute appendicitis in an ambulatory setting^27–30^. Therefore, to confirm the real prolonged LOS we used 2*75 IQR of the entire cohort (8 days). Only less than 5% had prolonged LOS. In our opinion, this period was associated with either postoperative complications or delayed recovery. In addition, we confirmed that conversion might contribute to prolonged LOS. This obvious fact has been extensively studied before in comparative studies and randomized controlled trials including LA and open procedure^31^. The reason for conversion was often severe inflammatory process, periappendiceal abscess or intraoperative adverse events. These may be the real cause of longer hospital stay, not the conversion itself. In the majority of cases such patients required longer antibiotic treatment or developed postoperative complications. So prolonged LOS was a consequence of these circumstances.

Patients who developed complications or required reintervention had higher rates of readmission. This fact should be taken under consideration when discharging such patients even if in good condition. On the contrary, complicated appendicitis was not a risk factor for readmission. Interestingly, patients operated by residents had a higher risk of readmission. In all centers participating in the study, hospital discharge is based on the decision of the attending surgeon, not the resident. We can exclude any erroneous assessment of the patient’s condition by residents before discharge. On the basis of our study we could not clearly identify any causal explanation for that finding. Nevertheless, Jolley *et al*. also concluded that the involvement of resident in operation was associated with higher risk of readmission (OR 1.54 (1.23–1.94)^32^. They were, however, not able to explain this phenomenon. On the other hand, Advani *et al*. reported lower risk of hospital readmission when the resident was involved in operation^33^. Other studies show that appendectomy performed by residents is not associated with more complications^34,35^. Even though whether residents performed LA is linked with readmission rates, it is still considered a perfect model for training in laparoscopic surgery^35^. Those contradictory results suggest that the evidence on the impact of residents in performing LA is rather weak and requires further investigation.

Our study does have certain limitations as in other observational multicenter reports. First, most of the patients were included retrospectively, rendering it impossible to analyze accurately each participating center’s perioperative protocol, such as antibiotic regimens or discharge criteria. As shown in other large observational studies, management of AA among surgical centers significantly varied^19^. The design of the study did not introduce any changes in the perioperative protocols in each participating centers. It is likely the surgical care, antibiotic regimens and perhaps even the technique differed in each center. We, however, believe it has little impact on final outcomes and allowed us to draw more universal conclusions. Second, the readmission rate in our data only represents readmissions to the original surgical department due to complications, instead of the overall rate that included readmissions to other departments or hospitals, which could underestimate the true readmission rate. The readmission statistics, however, are combined from all study groups, reducing bias. Third, the surgical procedures were performed by surgeons with different surgical skills. Subsequently, we did not differentiate between types of diabetes and we did not take into account complications related to diabetes. Although type 1 diabetes mellitus (T1DM) and type 2 diabetes mellitus (T2DM) have different backgrounds, the mechanism leading to complications (e.g. infectious) is relatively similar. Therefore, we have decided not to differentiate between types of DM. Lastly, in our study the diagnosis of complicated AA was based on intraoperative and pathological visualization of the appendix^36^. Imran *et al*. reported that even 40% of patients were incorrectly classified when comparing intraoperative image of the appendix to results of pathological examination^37^.

## Conclusions

Our study confirmed that laparoscopic appendectomy is a safe procedure associated with low rates of complications, prolonged LOS, and readmissions. We identified risk factors for those events such as complicated appendicitis, postoperative morbidity, conversion and reintervention after primary procedure. Occurrence of these events during treatment should alert the surgeons and help select patients that might require customized treatment to minimize the risk of these adverse events.

## Data Availability

The datasets used and/or analyzed during the current study are available from the corresponding author on reasonable request.
