# Author Correction: Risk factors for serious morbidity, prolonged length of stay and hospital readmission after laparoscopic appendectomy - results from Pol-LA (Polish Laparoscopic Appendectomy) multicenter large cohort study

**DOI:** 10.1038/s41598-019-54993-3

**Published:** 2019-12-06

**Authors:** Maciej Walędziak, Anna Lasek, Michał Wysocki, Michael Su, Maciej Bobowicz, Piotr Myśliwiec, Kamil Astapczyk, Mateusz Burdzel, Karolina Chruściel, Rafał Cygan, Wojciech Czubek, Natalia Dowgiałło-Wnukiewicz, Jakub Droś, Paula Franczak, Wacław Hołówko, Artur Kacprzyk, Wojciech Konrad Karcz, Jakub Kenig, Paweł Konrad, Arkadiusz Kopiejć, Adam Kot, Karolina Krakowska, Maciej Kukla, Agnieszka Leszko, Leszek Łozowski, Piotr Major, Wojciech Makarewicz, Paulina Malinowska-Torbicz, Maciej Matyja, Maciej Michalik, Adam Niekurzak, Damian Nowiński, Radomir Ostaszewski, Małgorzata Pabis, Małgorzata Polańska-Płachta, Mateusz Rubinkiewicz, Tomasz Stefura, Anna Stępień, Paweł Szabat, Rafał Śmiechowski, Sebastian Tomaszewski, Viktor von Ehrlich-Treuenstätt, Maciej Wasilczuk, Mateusz Wierdak, Anna Wojdyła, Jan Wojciech Wroński, Leszek Zwolakiewicz, Michał Pędziwiatr

**Affiliations:** 1 Department of General, Oncological, Metabolic and Thoracic Surgery, Military Institute of Medicine, Warsaw, Poland; 2 2nd Department of General Surgery, Jagiellonian University Medical College, Krakow, Poland; 3 Department of Surgical Oncology, Medical University of Gdansk, Gdansk, Poland; 4 1st Department of General and Endocrinological Surgery, Medical University of Bialystok, Bialystok, Poland; 5 2nd Department of General, Vascular and Oncological Surgery, Second Faculty of Medicine, Warsaw, Poland; 6 Department of General Surgery, SPZOZ in Węgrów, Węgrów, Poland; 7 Department of General, Oncological and Minimal Invasive Surgery, Żeromski’s General Hospital, Krakow, Poland; 8 Department of General, Minimally Invasive and Oncology Surgery, Regional Hospital named J.Śniadecki, Białystok, Poland; 9 Department of General, Minimally Invasive and Elderly Surgery, University of Warmia and Mazury in Olsztyn, Olsztyn, Poland; 10 Department of General and Oncological Surgery, Ceynowa Hospital, Wejherowo, Poland; 11 Department of General, Transplant and Liver Surgery, Medical University of Warsaw, Warszawa, Poland; 12 Clinic of General, Viscera and Transplantation Surgery, Ludwig Maximilian University, Munich, Germany; 13 Department of General, Oncologic and Geriatric Surgery, Jagiellonian University Medical College, Krakow, Poland; 14 Department of General Surgery and Surgical Oncology, Specialist Hospital in Kościerzyna, Kościerzyna, Poland; 15 Department of General, Oncological and Vascular Surgery, The Regional Subcarpathian John Paul II Hospital in Krosno, Krosno, Poland; 16 Clinical Department of General Surgery with Oncology, Gabriel Narutowicz Memorial City Specialty Hospital, Krakow, Poland; 17 Department of General and Laparoscopic Surgery, Municipal Hospital in Hajnówka, Hajnówka, Poland; 18 Department of General Surgery, Multispeciality Hospital in Nowa Sól, Nowa Sól, Poland; 19 Department of General and Minimally Invasive Surgery, Leczna Hospital, Leczna, Poland; 20 Department of General Surgery, Oncological Surgery and Chemotherapy, Dr Louis Błażek Memorial Hospital, Inowrocław, Poland; 21 Faculty of Health Sciences, Powiślańska School in Kwidzyn, Kwidzyn, Poland; 22 Emergency Department, Specialist Hospital in Kościerzyna, Kościerzyna, Poland

Correction to: *Scientific Reports* 10.1038/s41598-019-51172-2, published online 15 October 2019

The original version of this Article omitted an affiliation for Wojciech Makarewicz. The correct affiliations for Wojciech Makarewicz are listed below:

Department of Surgical Oncology, Medical University of Gdansk, Gdansk, Poland.

Department of General Surgery and Surgical Oncology, Specialist Hospital in Kościerzyna, Kościerzyna, Poland.

This has now been corrected in the HTML and PDF versions of this Article.

